# Chemotherapy diminishes lipid storage capacity of adipose tissue in a preclinical model of colon cancer

**DOI:** 10.1186/s12944-017-0638-8

**Published:** 2017-12-19

**Authors:** Maryam Ebadi, Catherine J. Field, Richard Lehner, Vera C. Mazurak

**Affiliations:** 1 Division of Human Nutrition, Department of Agricultural, Food and Nutritional Science, University of Alberta, Edmonton, AB Canada; 2 Department of Pediatrics, Group on Molecular and Cell Biology of Lipids, University of Alberta, Edmonton, AB Canada; 3 Faculty of Agricultural, Life & Environmental Science 4-002 Li Ka Shing Center for Research Innovation, University of Alberta, Edmonton, AB T6G 2E1 Canada

**Keywords:** Adipose atrophy, Cancer treatment, Lipogenesis, Lipid synthetic pathways, Fatty acids

## Abstract

**Background:**

Accelerated loss of adipose tissue in cancer is associated with shorter survival, and reduced quality of life. Evidence is emerging suggesting tumour association with alterations in adipose tissue, but much less is known about drug-related mechanisms contributing to adipose atrophy. Identification of mechanisms by which tumour and cancer treatments, such as chemotherapy, affect adipose tissue are required to develop appropriate therapeutic interventions to prevent fat depletion in cancer. This pre-clinical study aimed to assess alterations in adipose tissue during the clinical course of cancer.

**Methods:**

*Fischer 344* rats bearing the Ward colorectal tumour were euthanized before chemotherapy, after 1- cycle, or 2-cycles of a combination chemotherapy consisting of Irinotecan (CPT-11) combined with 5-fluorouracil (5-FU), which recapitulates first line treatment for human colorectal cancer. Periuterine adipose tissue was isolated. Healthy rats served as a reference group. Histological analysis (hematoxylin and eosin), Real-time PCR (TaqMan) and proteomic analysis (LC-MS/MS) were performed.

**Results:**

Larger adipocytes (3993.7 ± 52.6 μm^2^) in tumour-bearing animals compared to the reference group (3227.7 ± 36.7 μm^2^; *p* < 0.001) was associated with reduced expression of proteins involved in mitochondrial fatty acid oxidation. The presence of a tumour has a significant effect on phospholipid but not triglyceride fatty acid composition. There were greater proportions of saturated fatty acids concurrent with lower monounsaturated fatty acids within the PL fraction of adipocytes in tumour-bearing animals. Chemotherapy treatment decreased the size of adipocytes (2243.9 ± 30.4 μm^2^; *p* < 0.001) and led to depletion of n-3 polyunsaturated fatty acids in adipose tissue triglyceride. Evaluation of the proteome profile revealed decreased expression of proteins involved in ATP generation, β-oxidation, and lipogenesis. Overall, adipose tissue may not be able to efficiently oxidize fatty acids to provide energy to maintain energy demanding pathways like lipogenesis inside the tissue.

**Conclusions:**

In conclusion, metabolic adaptations to mitochondrial impairment may contribute to diminished lipid storage capacity of adipose tissue following chemotherapy delivery.

**Electronic supplementary material:**

The online version of this article (10.1186/s12944-017-0638-8) contains supplementary material, which is available to authorized users.

## Background

Human and animal studies have revealed adipose atrophy in a variety of cancer types [reviewed in [1]]. Accelerated loss of adipose tissue is associated with shorter survival, reduced quality of life, and impaired response to anti-cancer treatments [2–4]. On the other hand, studies have reported that adipose depletion may precede and occur more rapidly than muscle loss during cancer progression and that prevention of lipolysis prevents muscle loss in pre-clinical models [reviewed in [1]]. However, identification of molecular pathways by which the tumour and cancer treatments, such as chemotherapy, affect adipose tissue are required to develop appropriate therapeutic interventions to prevent fat depletion.

Mechanisms such as elevated lipolysis, decreased lipogenesis, impaired adipogenesis, elevated fat oxidation, and decreased lipid deposition contribute to adipose atrophy in cancer [1]. Although elevated lipolysis has been the focus of the majority of previous studies [5–10], adipose atrophy also occurs when lipogenesis is limited in white adipose tissue. Deterioration in lipid synthesizing capacity of adipose tissue, exemplified by decreased mRNA levels of key enzymes such as *acetyl-CoA carboxylase (ACC), fatty acid synthase (FAS), stearoyl-CoA desaturase-1 (SCD-1), lipoprotein lipase (LPL), diacylglycerol O-Acyltransferase-2 (DGAT-2)* as well as lower expression of *peroxisome proliferator-activated receptor gamma* (*PPARγ*), a key regulator of adipose tissue lipid metabolism, has been observed in previous studies [11–15].

A pre-clinical model of colorectal cancer has been developed to represent the same doses, cycles and levels of toxicity observed clinically in human colorectal cancer patients treated with Irinotecan (CPT-11) combined with 5-fluorouracil (5-FU) [16, 17], a first-line chemotherapy for patients with advanced colorectal cancer. This study aimed to assess the effect of a tumour and one or two cycles of chemotherapy drug delivery on periuterine adipose tissue morphology, fatty acid composition and expression of genes involved in lipogenesis in this pre-clinical model. To gain a global understanding of adipose tissue alterations in the neoplastic state, as well as to understand the effect of anti-neoplastic treatment on adipose tissue, proteomic analysis was performed after the second cycle of chemotherapy, a clinically relevant time point. We hypothesized that chemotherapy would decrease adipocyte size and expression of proteins involved in lipid synthesis pathways in adipose tissue. Given the contribution of adipose tissue in mediating whole body metabolism, identification of pathways mediating fat loss following chemotherapy treatment enhances understanding of aberrations of lipid metabolism in cancer and helps to define interventions to circumvent wasting.

## Methods

### Animal model and experimental design

All animal experiments were approved by the Institutional Animal Care Committee and performed in accordance with the Canadian Council on Animal Care Guidelines for the Care and Use of Laboratory Animals. Female *Fisher 344* rats (Charles River Laboratories, St. Constant, Quebec, Canada) were received at 150-180 g, 11 to 12 weeks of age and were held two per cage. All cages were kept in a room with controlled temperature (21–22 °C), a reverse light–dark cycle (12/12 h) and with food and water available ad libitum. Animals consumed a basal diet [Teklad TD.84172 basal mix with fat source omitted, Harlan Teklad, Madison, WI] to which we added different mixtures of commercially available fats to reach a 40% fat diet representative of the composition of western diets and many cancer patients. The polyunsaturated to saturated fat ratio in both diets was 0.35.

**Fig. 1:**
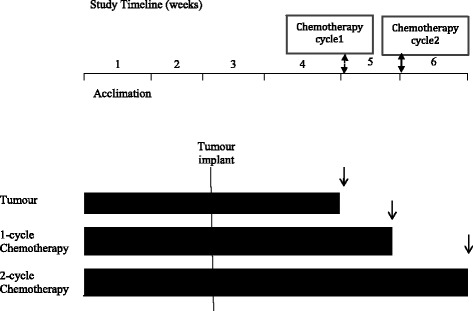
Experimental study design Reference animals did not undergo tumour injection or chemotherapy treatment but were handled similar to experimental groups. Tumour-bearing rats were injected with tumour, received no chemotherapy. Two weeks following implantation of the tumour, when the implants grew to approximately 2.3cm^3^ (or 1.2% of body weight), rats received CPT-11/ 5-FU combination regimens, for 1 or 2 cycles. The start day of chemotherapy cycle-1 (week 5); the first day of chemotherapy cycle-2 (week 6). ↓ End of each bar represents the day that animals were euthanized.

The experimental design in which rats were randomly assigned to 1 of 3 experimental groups is presented in Fig. 1. Rats serving as reference group (REF; *n* = 8) did not undergo tumour injection or chemotherapy treatment but were handled similar to the experimental groups. Additionally, there was a group of tumour-bearing rats (TUM; n = 8) that were injected with tumour, but received no chemotherapy. The Ward colorectal carcinoma, provided by Dr. Y. Rustum (Department of Cancer Biology, Chair, Director of Institute Core Resources, Roswell Park Cancer Institute, Buffalo, NY [18], was implanted subcutaneously in the left flank of the rats under light anaesthesia. The size of the tumour was measured as described previously [19].

Two weeks following implantation of the tumour, when the implants grew to approximately 2.3cm^3^ (or 1.2% of body weight), rats received CPT-11/ 5-FU combination regimens, for 1 or 2 cycles. The first cycle of chemotherapy (cycle 1) consisted of CPT-11 (50 mg/Kg BW/d s.c.) administered on day 0 and 5-FU (50 mg/Kg BW/d s.c) administered on day 1. The second chemotherapy cycle (cycle 2) consisted of the same drug regime occurring one week after cycle 1 (day 7 and 8). Subcutaneous injection of atropine (1 mg/kg) was given prior to each CPT-11. At study termination, animals were euthanized by carbon dioxide followed by decapitation. The periuterine adipose tissue was excised, weighed and immediately snap frozen in liquid nitrogen and stored at −80 °C for subsequent measurements or fixed in paraformaldehyde.

### Body weight and food intake

Body weight and food intake were measured every other day prior to chemotherapy initiation. After treatment initiation, animals were followed daily by measuring body weight and food intake. Body weight at baseline (day 0) was set to 100 and subsequent changes during chemotherapy were expressed as relative to the day 0 (%). Relative food intake during chemotherapy was reported as a percentage change of the baseline intake (the mean food intake prior to chemotherapy).

### Adipose tissue morphometry

Adipose tissue for microscopy was collected for reference, tumour-bearing and 1 and 2-cycles chemotherapy groups. Periuterine adipose tissue samples were fixed in 10% paraformaldehyde for 24 h, dehydrated in absolute ethanol, cleared in xylene then embedded in paraffin and cut into 5 μm sections. Sections were stained with Harris haematoxylin, counterstained with eosin, viewed at 20X magnification, and images were obtained with light microscopy (Olympus Qlmaging micropublisher camera). Adipocyte size was determined by measuring cross-sectional areas of 300 cells in 5 random fields from 5 rats/group using Image J software (National Institutes of Heath, alth, http://rsbweb.nih.gov/ij). A hemacytometer was used as a calibrator for measuring the size of adipocytes.

### Real-time-PCR

For RT-PCR, adipose tissue was collected from reference, tumour-bearing and 1 and 2-cycles chemotherapy groups. Total RNA was isolated from 50 mg ground tissue powder using an RNeasy Lipid Tissue Mini Kit (Qiagen) according to the manufacturer’s instructions and stored at −80 °C. RNA concentration was quantified spectrophotometrically (NanoDrop 1000; NanoDrop Technologies, Boston, MA), and the quality was assessed using the Agilent Bioanalyzer 2100 (Agilent Technologies, Palo Alto, CA, USA). Real-time (RT)-PCR was performed using Applied Biosystems (Foster City, CA, USA) reagents and instruments. All first-strand cDNA samples were synthesized from 750 ng total RNA per 20 μL reaction using the High Capacity cDNA reverse transcription kit (Applied Biosystems) on a GeneAmp PCR 9700 thermal cycler and then diluted 1:10 with nuclease-free water. PCR was performed with 10 μL TaqMan® Fast Advanced Master Mix (Life Technologies, Carlsbad, CA), 1 μL of a TaqMan® probe and primer set, 7 μL of NFH2O and 2 μL diluted cDNA in a 20 μl final reaction mixture. Pre-designed TaqMan® probes and primer sets (Life Technologies, Carlsbad, CA) with a 6-carboxyfluorescein phosphoramidite (FAM™) label on the 5′ end were used which contained the following assays: *fatty acid synthase* (Rn00569117_m1), *acetyl-CoA carboxylase alpha* (Rn00573474_m1), *stearoyl-Coenzyme A desaturase 1* (Rn00594894_g1), *peroxisome proliferator-activated receptor gamma* (Rn00440945_m1), *peroxisome proliferator-activated receptor gamma, coactivator 1 alpha* (Rn00580241_m1), *sterol regulatory element binding transcription factor 1* (*SREBP-1c*) (Rn01495769_m1), *ribosomal protein large P0* (Rn03302271gH), *diacylglycerol O-acyltransferase 2* (Rn01506787m1), *lipoprotein lipase* (Rn00561482_m1), *cell death-inducing DFFA-like effector a* (Rn04181355_m1). qRT-PCR reactions were amplified on an Applied Biosystems 7900HT Fast Real-Time PCR System using SDS 2.3 software for 10 min at 95 °C, followed by 95 °C for 15 s and 1 min at 60 °C for 40 cycles. Reactions were done in duplicate the relative target mRNA expression was described using the eq. 2^−ΔΔCT^ where ΔCT = (CT _target_ – CT_RPLP0_), thereby normalizing the data to the endogenous control mRNA of *RPLP0* [20].

### Proteomics

For proteomics analysis, the experiment was conducted on animals from reference, tumour and 2-cycles chemotherapy groups (*n* = 3). Frozen periuterine adipose tissue was ground in liquid nitrogen, mixed with in 10XRIPA lysis buffer (Pierce Biotechnology, Rockford, IL, USA), supplemented with protease and phosphatase inhibitors (Invitrogen Corporation, Frederick, MD, USA) in a 1:3 ratio for homogenization. The homogenate was then centrifuged at 12000 g for 20 min at 4 °C and the supernatant fraction was stored at −80 °C prior to analysis. Protein concentration was quantified using Pierce bicinchoninic acid (BCA) Protein Assay (Thermo Scientific, Rockford, IL, USA). Tissue homogenate was standardized for protein content based on the sample with the lowest protein content and 95 micrograms of total protein, in duplicates (repeated loading of the same sample), were loaded on 4–20% Mini-PROTEAN® TGX™ Precast Protein Gels (Bio-Rad, Hercules, CA). Digested gel lanes were analyzed using liquid chromatography-mass spectrometry (LC-MS/MS) at the Alberta Proteomics and Mass Spectrometry Facility (APM).

Briefly, excised gel bands were de-stained twice in ammonium bicarbonate/acetonitrile (ACN) (50:50), reduced (10 mM β–mercaptoethanol 100 mM AmBic), alkalated and digested with trypsin. Following trypsin digestion (6 ng/ul, 16 h, RT), peptides were extracted from the gel firstly using 97% water/2% ACN containing 1% formic acid and secondly with a 1:1 mixture of extraction buffer and acetonitrile. Nanoflow HPLC (Easy-nLC II, Thermo Scientific) coupled to the LTQ XL-Orbitrap hybrid mass spectrometer (Thermo Scientific) was used to ionized the tryptic peptides resolved in 25% ACN and 1% *v*/v formic acid. Peptide mixtures injection flow rate was 3000 nL/min, with the resolved rate kept at 500 nL/min using 60 min linear acetonitrile gradients from 0% to 45% v/v aqueous acetonitrile in 0.2% v/v formic acid. Data were obtained in a data-dependent manner in the Orbitrap spectra with a resolution of 60,000 and the collision induced dissociation was used to fragment ten most intense multiply charged ions, recorded in the linear ion trap. Data were analyzed by Proteome Discoverer 1.3 (Thermo Scientific) and searched using SEQUEST (Thermo Scientific) against the *Rattus norvegicus* protein database. A precursor mass tolerance of 10 ppm and a fragment mass tolerance of 0.8 Da were considered in search parameters. Data were normalized to the house-keeping protein, actin before statistical data analysis.

### Ingenuity pathway analysis (IPA)

IPA is a web-based analysis tool that enables identification of canonical pathways, biological processes, molecular and cellular functions, molecular networks, upstream regulators in a set of molecules of interest. Differentially expressed proteins (>1.5 fold change with a *p*-value <0.05) were uploaded into IPA software (Ingenuity Systems; Mountain View, CA, USA) and the significant canonical pathways, molecular and cellular functions, and upstream regulators were generated based on the known data in the literature. The significance was calculated using Fisher Exact test with the *p*-value <0.05.

### Adipose tissue fatty acid analysis

Frozen adipose tissue was ground in liquid nitrogen using mortar and pestle until crushed into a fine powder. Lipids were extracted using modification of the Folch procedure [21], by adding chloroform/methanol (2:1,vol/vol). Thin layer chromatography was used to isolate triglyceride and phospholipids, followed by saponification and methylation for triglyceride (TG) and direct methylation for phospholipid (PL) containing tubes. Fatty acid methyl esters were separated by an automated gas-liquid chromatograph (Vista 8400 autosampler, Varian CP-3400). The system used a bonded phase fused silica capillary column, BP20:25 mm X 0.25 OD SGE product. Helium was used as the carrier gas at a flow rate of 2.6 ml/min using a splitless injector. These conditions separate saturated, monounsaturated and polyunsaturated fatty acids from 12 to 24 carbon chain lengths by comparison with known standards. Proportions of saturated (SFA), monounsaturated (MUFA), n-6 and n-3 fatty acids were calculated.

### Statistical analysis

Data were reported as mean ± SEM and parametric or non-parametric statistic tests were chosen according to data distribution. One-way ANOVA with Bonferroni post-hoc comparisons was used to compare between groups when data was normally distributed. Non-parametric data were analyzed by Kruskal-Wallis non-parametric test followed by Dunn’s Multiple comparison test. The chemotherapy groups are tumor bearing animals, and reflect the impact of chemotherapy on a metabolic system already compromised by a tumor. Therefore Tumour group is compared to the reference, followed by comparing chemotherapy animals to the tumour as chemotherapy is administrated in tumour bearing state. A repeated measure ANOVA (repeated measure on food intake and body weight following chemotherapy initiation) was performed. Statistical analyses were performed using SPSS (SPSS for Windows, version 22.0, SPSS, Chicago, IL) and a difference was considered to be statistically significant if the *p* value was less than 0.05.

## Results

### Food intake and body weight

**Fig. 2:**
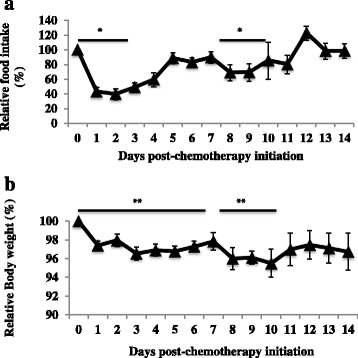
Relative **a**) food intake and **b**) body weight compared to the baseline during chemotherapy Values represent mean ± SEM. The x-axis represents days after chemotherapy initiation. Day 0 represents the first day of the first cycle of chemotherapy (*n* = 14), and day 7, representing the first day of the second cycle of chemotherapy (*n* = 7). Significant differences between day 0 and days 1–3 of the first cycle or between day 7 and days 8–10 of the second cycle of chemotherapy evaluated by repeated measure ANOVA (*p* < 0.05). **Significant difference between day 0 (baseline) and days 1–6 or between day 7 and days 8–10 following chemotherapy initiation evaluated by repeated measure ANOVA (*p* < 0.05).

Food consumption did not differ between reference (10 ± 0.4 g/d) and tumour bearing animals (9 ± 0.4 g/d; *p* = 0.1). Following the first day of each cycle of chemotherapy, food intake decreased in all groups, returning to baseline by the end of the cycle (Fig. 2a). Tumour-free body weight was reduced during chemotherapy cycles (Fig. 2b). Following the first day of the first cycle of chemotherapy, a decrease in body weight from baseline (Fig. 2b) was observed. The same pattern of weight loss was observed during the second cycle but with a greater decrease in body weight during the first 3 days post chemotherapy initiation. Percent change of body weight from baseline at day 1 (the second day of the first cycle) was −2.6% ± 0.4 and after the second cycle (day 8) was −4.0% ± 1.2.

### Adipose tissue weight and histological characteristics

**Fig. 3:**
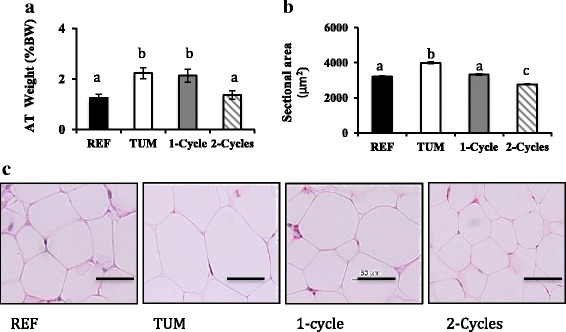
Periuterine adipose tissue weight and morphological characteristics. **a**) Periuterine adipose tissue weight (%BW), **b**) Morphometric analysis of adipocyte cross sectional area (μm^2^) in *Fischer 344* rats, **c**) Example images of periuterine adipocytes stained with hematoxylin and eosin (magnification 20X) from Reference, Tumour, and chemotherapy groups. Values are mean ± SEM, different superscripts (a, b, c) indicate significant differences (*p* < 0.05) determined by Kruskal–Wallis test for periuterine adipose tissue weight (n = 7–8/group) and one way ANOVA for cross sectional area (1500 cells/group; 5 animals/group). Bar = 50 μm. Abbreviations: REF, Reference; TUM, Tumour

Periuterine adipose tissue weight, as a percentage of tumour free body weight, was significantly higher in the tumour (*p* = 0.001) group compared to the reference group. No change in adipose tissue weight was observed after 1 cycle of chemotherapy. However, following 2 cycles of chemotherapy, adipose tissue weight declined and was lower than the tumour group (*p* = 0.005; Fig. 3a).

We examined whether the reduction in periuterine adipose mass following chemotherapy was detectable at the microscopic level. Examples of adipocytes stained with hematoxylin and eosin (H&E) from reference, tumour, and chemotherapy groups are presented in Fig. 3c. Adipocytes were surrounded with a thin rim of cytoplasm in which nuclei are compressed into the peripheral rim. Larger adipocytes, determined by cell cross sectional area, were observed in tumour group compared to the other groups (Fig. 3b). Tumour-bearing animals (3993.7 ± 52.6 μm^2^) had larger adipocytes than the reference group (3227.7 ± 36.7 μm^2^, *p* < 0.001). Chemotherapy decreased the size of adipocytes after the first cycle with greater reduction during the second cycle of chemotherapy (Fig. 3b).

### mRNA expression of genes involved in lipid metabolism in adipose tissue

**Fig. 4:**
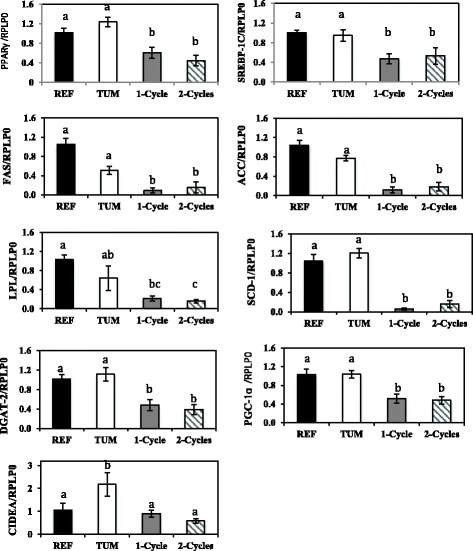
Relative mRNA levels of genes encoding various lipogenic enzymes assessed using Real-time PCR, The mRNA levels of the target genes included *FAS, ACC, SCD-1, DGAT-2* as well as *LPL*, *PPARγ, SREBP-1c, PGC-1α* and *CIDEA* were normalized to the expression of *RPLP0* and are shown as mean ± SEM. Results are fold change of gene expression relative to the reference group; different superscripts (a, b, c) indicate significant differences (*P* < 0.05) determined by Kruskal–Wallis Test; (*n* = 6–8/group). Abbreviations: REF, Reference; TUM, Tumour

mRNA expression of several key genes involved in lipogenesis as well as major transcriptional factors expressed in adipose tissue including *PPARγ*, *SREBP-1c* and *PGC-1α* were assessed. The tumour alone had no significant effect on the expression of transcriptional factors nor the genes regulating lipogenic proteins. However, mRNA expression of *CIDEA*, a lipid droplet-associated protein, was 2-fold higher in tumour-bearing animals compared to the reference animals (Fig. 4). After the first cycle of chemotherapy, expression of all genes assessed were decreased, with the exception of *LPL* which was significantly decreased only after cycle 2. Expression of all genes remained significantly lower after the second cycle compared to the tumour-bearing animals (Fig. 4).

### Protein expression in adipose tissue

We first evaluated the influence of the tumour on global protein expression in adipose tissue. In general, proteins involved in fatty acid β-oxidation such as *hydroxysteroid (17-beta) dehydrogenase 10 (HSD17B10), enoyl-CoA hydratase, short chain, 1, mitochondrial (ECHS1), enoyl-CoA delta isomerase 1 (ECI1)* were down- regulated in tumour-bearing animals (*p*-value < 0.001, Additional file 1). Expression of *protein kinase cAMP-activated catalytic subunit alpha (PRKACA)*, involved in phosphorylation and activation of various proteins such as *hormone sensitive lipase* (*HSL*), was decreased by 6 fold. Expression of two transporters, *ATP binding cassette subfamily D member 2* (*ABCD2*), involved in transport of very long chain acyl-CoA into peroxisomes and *sterol carrier protein 2 (SCP-2)*, responsible for fatty acid and phospholipid transport and involved in oxidation of very long chain fatty acids in peroxisomes were up regulated (6 and 2 fold) compared to the reference animals.

Next, we evaluated the impact of chemotherapy on adipose tissue protein expression. Similar to what was observed for gene expression, major changes in protein expression occurred after chemotherapy treatment (Additional file 2). A total of 121 proteins were differentially expressed in periuterine adipose tissue after 2 cycles of chemotherapy compared to the tumour. Of these proteins, 112 were down-regulated whereas 12 proteins were up-regulated. Mapping each protein accession number to IPA identified associated canonical pathways. The top canonical pathways identified are presented in Table 1. Overall, chemotherapy induced alterations in mitochondria-linked pathways involved in glucose and lipid metabolism. Mitochondrial dysfunction was manifested by reduction in proteins mainly involved in ATP production. Reduced ATP production may result from inhibition of protein complexes involved in oxidative phosphorylation such as *ATP synthase subunits, succinate dehydrogenase (SDH)*, *NADH dehydrogenase (ubiquinone) subunits (NDUFs), Coenzyme Q – cytochrome c reductase subunits (UQCRC1, UQCRC2, UQCRFS1)* and *pyruvate dehydrogenase (PDH)*. Low levels of *pyruvate dehydrogenase complex (PDHC)* would be expected to reduce acetyl-CoA biosynthesis. Moreover, there was a reduction in expression of proteins involved in mitochondrial antioxidant systems such as *cytochrome c oxidase (COX)*, and reactive oxygen species scavenging enzymes such as *peroxiredoxin (PRDX), glutathione S-transferase* and *catalase (CAT)* (Table 1).

**Table 1:** Top Canonical pathways identified in adipose tissue of rats undergoing two cycles of chemotherapy

Pathway	*P*-value	Ratio	Molecules
Mitochondrial Dysfunction	5.01E-14	0.1	*SDHB, NDUFA9, ATP5O, MT-CO2, PDHA1, PRDX3, PARK7, NDUFV2, UQCRC2,CAT, COX5A, UQCRFS1, VDAC1,UQCRC1, MAOA, GPX3, GSTMS*
Gluconeogenesis	7.94E-13	0.3	*ENO1, ENO3, PGAM1, ALDOA, GAPDH, ME1,* *MDH2, ALDOC*
Glycolysis	4.90E-09	0.2	*ENO1, ENO3, PGAM1, ALDOA, GAPDH, ALDOC*
Oxidative Phosphorylation	1.26E-08	0.1	*SDHB, NDUFA9, NDUFV2, UQCRC2, ATP5O, COX5A, UQCRFS1, MT-CO2, UQCRC1*
Krebs Cycle	1.74E-07	0.2	*SDHB, CS, IDH3A, IDH2, FH, MDH2*
Acetyl-CoA Biosynthesis (Pyruvate Dehydrogenase Complex)	6.31E-06	0.4	*PDHA1, DLAT, PDHB*
TR/RXR Activation	2.09E-05	0.1	*ENO1, FAS, ACACA, PCK1, ME1, THRSP*
Pentose Phosphate Pathway	2.09E-05	0.3	*PGD, TKT, PGLS*
Fatty Acid β-oxidation	3.24E-05	0.1	*ECHS1, ACAA1, SCP2, IVD, CPT2, ACADVL*

The ratio is calculated based on the numbers of proteins in a given pathway divided by total numbers of proteins that make up that pathway in IPA. *P*-value calculated by Fisher’s exact test. Abbreviations: *TR* Thyroid hormone receptor*, RXR* Retinoid X receptor*, LXR* Liver X receptor

Proteins involved in pathways related to fatty acid metabolism including fatty acid biosynthesis (*ACYL, FAS, ACC*) and fatty acid β-oxidation were down regulated. *LXR* target proteins, *ACC* and *FAS* were down regulated by 7 and 8 fold respectively after 2 cycles of chemotherapy compared to the tumour group. The expression of *glycerol-3-phosphate dehydrogenase (GPD1)* was also suppressed. *Cytoplasmic GPD1* connects glycolysis to lipid biosynthesis by converting dihydroxyacetone phosphate to glycerol-3-phosphate. There was a reduction in the expression of enzymes that generates NADPH for fatty acid biosynthesis including *glucose-6-phosphate dehydrogenase (G6PD), phosphogluconate dehydrogenase (PGD), malate dehydrogenase 2 (MDH2), Isocitrate dehydrogenase (IDH)*. On the other hand, enzymes implicated in fatty acid β-oxidation such as *carnitine palmitoyltransferase 2 (CPT2), acetyl-CoA acyltransferase 1 (ACAA1), enoyl-CoA hydratase (ECH), enoyl-CoA delta isomerase 1 (ECl1), acyl-CoA dehydrogenase, C-4 to C-12 straight chain (ACADM)* and *acyl-CoA dehydrogenase*, and *very long chain (VLCAD)* were also down regulated. Chemotherapy also caused reduction in protein expression of other molecules including *hormone-sensitive lipase (HSL), solute carrier family 2 member 4 [SLC2A4 (GlUT4)]*, *solute carrier family 25 Member 1 (SLC25A1)*, involved in citrate transport across inner mitochondrial membrane and *mitochondrial pyruvate carrier 2 (MCP2)*, necessary for pyruvate transport across a mitochondrial membrane. Molecules in other major metabolic pathways including glycolysis, pentose phosphate pathway, gluconeogenesis were also down-regulated (Table 1). Top highly activated and inhibited upstream regulators predicted by IPA following 2 cycles of chemotherapy are summarized in Table 2. The 5-top upstream regulators predicted to be inhibited were *PGC-1α, PPARγ, PPARα*, *MYC* and *SERBP-1c*. The chemical drug, 5-FU, was predicted to be activated with highly significant positive z score.

**Table 2:** Activated and inhibited upstream regulators predicted by IPA following 2 cycles of chemotherapy

Upstream regulator	Molecular type	Predicted activation state	Activation Z-score*	*P*-value of overlap	Target molecules in differentially expressed dataset
*PPARGC1A*	Transcription regulator	Inhibited	−4.379	2.17E-19	*ACACA, ACADVL, ACAT1, ATP5O, CAT, COX5A, CS,DLAT, FASN, IDH3A, MDH2, ME1, MT-CO2, NDUFV2, PCK1, PDHA1, PGAM1, PRDX3, SLC2A4,UQCRFS1*
*PPARγ*	Ligand-dependent nuclear receptor	Inhibited	−4.06	1.629E-23	*ACAA1, ACACA, ACLY, ATP5O, CAT, CPT2, CS, DLAT, FABP1, FASN, FDPS, GAPDH, GPD1,GPT, IDH1, IVD, KRT18, LIPE, ME1, MGLL, PC, PCK1, PDHB, PYGL, RPSA, SCP2, Slc25a1, SLC2, TKT*
*PPARα*	Ligand-dependent nuclear receptor	Inhibited	−2.97	4.41E-16	*ACAA1, ACACA, ACADVL, ACAT1, ACAT2, CAT, CPT2, CS, DBI, FABP1, FASN, FDPS, GPD1, GPT, MGLL,MT-CO2, PC, PCK1, PRDX6, SCP2, SLC2A4, UQCRC1*
*MYC*	Transcription regulator	Inhibited	−2.61	2.27E-12	*ACACA, ACAT1, AK2, ALDOA, CANX, CAPNS1, CPT2, DBI, ENO1, FABP1, FASN, GAPDH, GCSH, GPT, HSPE1, IDH1, IDH2, LUM, NDRG2, PCK1, PDHA1, PGAM1, PHB, PHB2, PRDX3, SLC25A5, TKT*
*SREBF1*	Transcription regulator	Inhibited	−2.13	7.82E-09	*ACACA, ACLY, ALDOC, DBI, FASN, FDPS, GPX3, IDH1, PCK1, THRSP, UQCRFS1*
*MAP4K4*	Kinase	Activated	3.44	8.13 E-13	*ACACA, ACADVL, ACLY, DLAT, FASN, IVD, MGLL, PGAM1, SCP2, SLC2A4, UQCRC1, UQCRFS1*
5-Fluorouracil	Chemical drug	Activated	3.21	1.30E-11	*ALDOA, ATP5O, CANX, CAPNS1, ECHS1, FDPS, GAPDH, HSPE1, IDH2, NDUFV2, PSMA7, RPS8, SLC25A5, UQCRC2*

*Z > 2 and Z < −2 predict activation and inhibition of the upstream regulator, respectively. The p-value indicates the significance of the overlap between the molecules targeted by the upstream regulator in the IPA database and the experimental dataset. Abbreviations: *PPARγ, Peroxisome proliferator-activated receptor gamma; PPARα, Peroxisome proliferator-activated receptor alpha; PPARGC1A, Peroxisome proliferator-activated receptor gamma coactivator 1-alpha; SREBF1 (SREBP1c), Sterol regulatory element binding transcription factor 1; MAP4K4, Mitogen-activated protein kinase kinase kinase kinas*

### Fatty acid composition of periuterine adipose tissue

#### Triglyceride fatty acids

The fatty acid composition of TGs in periuterine adipose tissue are shown in Table 3. The most abundant fatty acids in TGs fraction were 18:1n-9 > 18:2n-6 > 16:0 > 18:0. The tumour had no effect on TG fatty acid composition of AT in comparison to the reference group. Animals that received 2 cycle of chemotherapy had lower proportions of 16:0 and 16:1 compared to the tumour group. Chemotherapy resulted in a significant decline of 18:3 n-3 proportions in the AT after each cycle of chemotherapy, compared to the tumour group. Proportion of total n-3 PUFAs, was significantly lower after both 1 and 2 cycles of chemotherapy compared to the tumour group, resulting in a significant increase in the ratio of n-6/n-3 fatty acids (*p* < 0.001).

**Table 3:** Fatty acid composition of triglyceride in periuterine adipose tissue of Fischer 344 rats

Fatty acids (% total)	REF	TUM	1- Cycle 2- Cycles	
C16:0	20.6 ± 0.5^a^	19.7 ± 0.3^a^	20.0 ± 0.4^a^	17.9 ± 0.7^b^
C16:1	3.9 ± 0.2^a^	3.6 ± 0.1^a^	3.4 ± 0.2^a^	2.6 ± 0.3^b^
C18:0	6.0 ± 0.3^a^	6.9 ± 0.2^a^	6.7 ± 0.1^a^	7.9 ± 0.4^a^
C18:1	41.3 ± 2.1^a^	42 ± 1^a^	41.3 ± 0.6^a^	44.3 ± 0.9^a^
C18:2n6	22.3 ± 1.6^a^	22.0 ± 0.7^a^	23.4 ± 0.5^a^	22.5 ± 0.4^a^
C18:3n3	1.6 ± 0.1^a^	1.7 ± 0^a^	1.3 ± 0^b^	1.2 ± 0.1^b^
C20:4n6	0.8 ± 0.1^a^	0.7 ± 0^a^	0.6 ± 0^a^	0.6 ± 0^a^
C20:5n3	ND	ND	ND	ND
C22:6n3	0.4 ± 0.1^a^	0.4 ± 0^a^	0.3 ± 0^a^	0.3 ± 0^a^
ΣSFA	28.3 ± 0.3^a^	28.2 ± 0.2^a^	28.3 ± 0.3^a^	27.3 ± 0.3^a^
ΣMUFA	45.7 ± 2^a^	46.1 ± 1^a^	45.2 ± 0.6^a^	47.4 ± 0.7^a^
Σn-6/n-3	11.2 ± 0.6^a^	10.3 ± 0.2^a^	13.6 ± 0.5^b^	14.7 ± 0.7^b^
Σn-6	23.8 ± 1.7^a^	23.4 ± 0.8^a^	24.7 ± 0.5^a^	23.7 ± 0.4^a^
Σn-3	2.2 ± 0.3^a^	2.3 ± 0.1^a^	1.8 ± 0.1^b^	1.6 ± 0.1^b^

Triglyceride fatty acids in periuterine adipose tissue of Fischer 344 rats bearing the Ward colorectal carcinoma receiving 1- or 2- cycles of chemotherapy. Healthy rats were used a reference for comparison (REF). Mean ± SEM, Kruskall test was used to determine significant differences between groups. Different superscripts (a, b) indicate significant differences between groups (*P* < 0.05); (*n* = 6–8/group). Abbreviations: *SFA*, saturated fatty acids; *MUFA*, monounsaturated fatty acids; *PUFA*, polyunsaturatsed fatty acids; *REF*, Reference; *TUM*, Tumour-bearing; *ND*, not determined

#### Phospholipid fatty acids

The fatty acid composition of PLs in periuterine adipose tissue is shown in Table 4. The presence of a tumour increased the proportions of C18:0 and total SFAs and decreased proportions of MUFAs (C16:1, C18:1) and C18:3n-3 in PLs which did not change after either 1 and 2 cycles of chemotherapy. No significant change in total n-3 PUFAs was observed after chemotherapy.

**Table 4:** Fatty acid composition of phospholipids in periuterine adipose tissue of Fischer 344 rats

Fatty acids (% total)	REF	TUM	1- Cycle 2-Cycles	
C16:0	26.4 ± 1.4^a^	26.2 ± 2^a^	28.8 ± 1.3^a^	29.4 ± 1.4^a^
C16:1	3.0 ± 0.1^a^	1.6 ± 0.2^b^	1.4 ± 0.1 ^b^	1.5 ± 0.2^b^
C18:0	22.3 ± 0.6^a^	30.3 ± 1.1^b^	29.2 ± 1.1^b^	30.1 ± 1.4^b^
C18:1	26.3 ± 1.4^a^	17.0 ± 1.5^b^	16.9 ± 1.5^b^	16.1 ± 1.2^b^
C18:2n6	13.4 ± 0.6^a^	12.4 ± 0.8^a^	12.2 ± 0.8^a^	11.6 ± 0.6^a^
C18:3n3	0.8 ± 0 ^a^	0.2 ± 0^b^	0.2 ± 0.1^b^	0.2 ± 0^b^
C20:4n6	5.4 ± 0.6^a^	8.3 ± 0.7^a^	7.6 ± 0.5^a^	7.03 ± 0.5^a^
C20:5n3	0.1 ± 0^a^	ND^a^	ND^a^	ND^a^
C22:6n3	0.8 ± 0.1^a^	0.5 ± 0^ab^	0.3 ± 0^b^	0.3 ± 0^b^
ΣSFA	49.9 ± 1^a^	57.9 ± 2.3^b^	59.2 ± 1.8^b^	61 ± 1.9^b^
ΣMUFA	29.4 ± 1.3^a^	19.2 ± 1.6^b^	19.2 ± 1.6^b^	18.3 ± 1.4^b^
Σn-6/n-3	11.1 ± 0.5^a^	16.1 ± 0.5^ab^	21.0 ± 1.6^b^	18.6 ± 1.2^b^
Σn-6	18.9 ± 0.6^a^	21.5 ± 1.1^a^	20.6 ± 0.8^a^	19.5 ± 0.8^a^
Σn-3	1.7 ± 0.1^a^	1.3 ± 0.1^ab^	1.0 ± 0.1^b^	1.1 ± 0.1^b^

Phospholipid fatty acids in periuterine adipose tissue of Fischer 344 rats bearing the Ward colorectal carcinoma receiving 1- or 2- cycles of chemotherapy. Healthy rats were used a reference for comparison (REF). Mean ± SEM, Kruskall test was used to determine significant differences between groups. Different superscripts (a, b) indicate significant differences between groups (*P* < 0.05); (n = 6–8/group). Abbreviations: *SFA* saturated fatty acids, *MUFA* monounsaturated fatty acids, *PUFA* polyunsaturated fatty acids; REF, Healthy, *TUM* Tumour-bearing, *ND* not determined

## Discussion

**Fig. 5:**
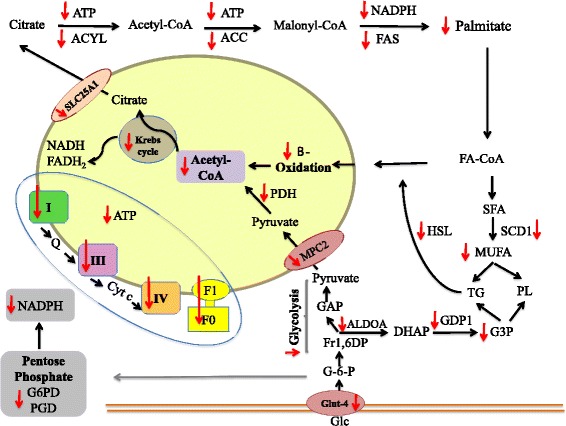
Schematic diagram summarizing adipose tissue alterations in rats undergoing 2-cycles of chemotherapy. Expression of many proteins within adipose tissue are altered in response to chemotherapy, which are mainly related to mitochondrial function. Mitochondrial dysfunction associates with decreased expression of proteins involved in ATP generation, fatty acid β-oxidation, Krebs cycle and acetyl-CoA production inside mitochondria. There was also a reduction in the expression of proteins in other metabolic pathways such as glycolysis, pentose phosphate, lipogenesis as well as decreased glucose uptake. Red arrows indicates reduced expression of proteins. Abbreviations: GLUT4, Glucose transporter-4; G-6-P, Glucose 6-phosphate; Fr 1,6-DP, Fructose 1,6-diphospate; GAP, Glyceraldehyde3-P; DHAP, Dihydroxyacetone phosphate; GDP1’ Glyceraldehyde-3-phosphate dehydrogenase; G3P, Glycerol-3-phosphate; TG, triglyceride; PL, Phospholipid; MUFA, Monounsaturated fatty acid; SCD1, Stearoyl-coenzyme A desaturase 1; FA, Fatty acid; HSL, Hormone-sensitive lipase; MPC2, Mitochondrial pyruvate carrier 2; SLC25A1, Solute carrier family 25 member 1; G6PD, Glucose-6-phosphate dehydrogenase; PGD, 6- phosphogluconate dehydrogenase; ACYL, ATP citrate lyase; ACC, Acetyl-coenzyme A carboxylase alpha; FAS, Fatty acid synthase

Lipid metabolism in the neoplastic state has been minimally investigated and even less is known about the effect of cytotoxic drugs, which may also induce alterations in adipose tissue. The current study investigated the effect of a tumour and sequential delivery of first line chemotherapy (5-FU + CPT-11) on measures of periuterine adipose tissue lipid metabolism in a pre-clinical model resembling the clinical course for humans with colorectal cancer. Based on observations from tumour-bearing animals and those receiving chemotherapy, we propose that inhibition of proteins involved in mitochondrial fatty acid oxidation and decreased HSL expression involved in lipolysis contributed to larger adipocytes in tumour-bearing animals, whereas lower expression of proteins involved in fatty acid synthesis and re-esterification concurrent with down regulation of proteins in mitochondria-linked metabolic pathways explain the observation that adipocytes are smaller in size after chemotherapy treatment. Figure 5 summarizes altered protein expression in adipose tissue in rats undergoing two-cycles of chemotherapy.

Protein identification by LC-MS-based quantitative proteomics enables comprehensive elucidation of protein expression and associated pathways that might be altered in health and disease [22]. Protein expression profiling in this study was a practical approach to determine crucial metabolic pathways altered in adipose tissue in the presence of tumour and anti-cancer treatments. The larger adipocytes observed in the tumour-bearing animals could be explained by the proteomics data which revealed inhibition of proteins involved in fatty acid β-oxidation and *HSL*-mediated lipolysis. Elevated *CIDEA* expression and diminished *PRKACA*, which phosphorylates and activates HSL, suggests that lipolysis was inhibited in tumour-bearing animals. This finding is consistent with previous studies reporting larger lipid droplets, diminished lipolysis and elevated triglyceride storage capacity of adipose tissue concurrent with *CIDEA* overexpression in adipose tissue [23, 24]. It should be noted that depending on the time (stage) of tumour growth and type of adipose tissue, previous studies have reported either smaller or larger adipocytes in tumour-bearing animals [25, 26]. Smaller epididymal adipocytes but larger mesenteric adipocytes were observed in rats bearing the Walker 256 carcinoma 2 weeks after tumour implantation [26].

The presence of a tumour has a significant effect on PL but not TG fatty acid composition. There were greater proportions of SFAs and reduction in MUFAs within the PL fraction of adipocytes. Elevated transportation of very long chain acyl-CoA into peroxisomes, evident by elevated protein expression of ABCD2 and SCP2, involved in oxidation of very long chain fatty acids, concurrent with inhibited expression of proteins involved in fatty acid β-oxidation may lead to the greater proportion of saturated fatty acid. This finding is in line is in line with previous studies indicating accumulation of saturated fatty acids when mitochondrial fatty acid oxidation enzymes are deficient [27]. Changes in membrane PL composition alter membrane fluidity and affects physiological functions such as glucose transport, insulin signalling and membrane-bound enzymes activity, transporters, and receptors [28, 29].

Diminished expression of proteins involved in Krebs cycle associates with down-regulation of pathways involved in the generation of substrates for Krebs cycle. Acetyl-CoA produced by fatty acid β-oxidation or derived from pyruvate (glycolysis) in mitochondria is a source for fatty acid synthesis [30]. In this study, lower expression of proteins in pathways involved in acetyl-CoA production was observed and consequently, a reduction in lipogenic enzymes protein expression such as *FAS* and *ACC*. Our results are also consistent with previous studies reporting decreased expression of *PPARγ, SREBP-1c, C/EBPα, FAS,* and *LPL* concurrent with diminished triglyceride content of adipocytes by inhibition of mitochondrial oxidative phosphorylation (OXPHOS) activity [31]. On the other hand, mitochondrial impairment associates with decline in oxidative phosphorylation and ATP production [reviewed in [32]]. ATP produced in mitochondria is necessary for the energy demanding lipogenic pathway [31]. De novo lipogenesis is one of the major pathways that could affect adipocyte composition and size. There was a reduction in expression of lipogenic enzymes such as *ACYL, ACC, FAS* and *SCD-1* in rats receiving chemotherapy; rats with lower adipose tissue expression of lipogenic genes exhibited lower proportions of palimitate, the primary product of FAS, in stored TGs.

Chemotherapy decreased food intake for 3 days; however, food intake was recovered by the end of each cycle. Reduced food intake and glucose availability associates with elevated fatty acid β-oxidation as an adaptive response to negative energy balance [33, 34]. In this study, however, we observed lower expression of proteins involved in fatty acid β-oxidation, therefore, factors other than food intake, contribute to the observed alterations. Reduced *HSL* protein expression and subsequently, decreased fatty acid availability may reduce substrate available for not only mitochondrial fatty acid β-oxidation but also as a ligand for *PPARγ*. Reduced *PPARγ* expression was associated with lower expression of *PPARγ* -target genes (*ACC, FAS, SCD-1*). Further studies are required to elucidate whether *PPARγ* agonists may help to maintain chemotherapy-altered adipose tissue metabolism. On the other hand, reduced HSL expression might be the consequence of lower mitochondrial β-oxidation. Whether decreased lipogenic protein expression and lipid storage capacity of adipose tissue by chemotherapy is a compensatory mechanism for decreased *HSL* expression, or is the consequence of mitochondrial impairment and inhibited mitochondria-linked pathways, remains uncertain. However, due to impaired OXPHOS and reduced *PGC-1α* mediated mitochondrial biogenesis, would suggest mitochondrial dysfunction as a plausible explanation. Thus, it seems that reduction of proteins in pathways such as glycolysis and lipolysis that produce substrate (pyruvate, fatty acids) for mitochondria compensates for mitochondrial dysfunction.

Mitochondrial damage in tumour cells by 5-FU [35] and mitochondrial membrane interruption by CPT-11/5-FU in colon cancer cell lines [36] have been reported in previous studies. Therefore, it would seem that this drug combination also has capacity to evoke these alterations in tissues other than the tumour. Alterations in adipose tissue mitochondrial function and related lipid metabolic pathways may associate with impaired lipid storage capacity of tissue, resulting in ectopic fat accumulation in peripheral tissues and consequently, exacerbate the dysregulated whole body lipid and glucose metabolism. Although diminished expression of proteins involved in mitochondrial function, lipogenesis and β-oxidation are occurring as a consequence of chemotherapy treatment and contributing to diminished size of adipocytes, many details remain to be explored.

The novel aspect of our study is that it is the first to report potential pathways evoking fat loss in a pre-clinical model of cancer during treatment with first line chemotherapy agents. We would like to emphasize that this model is not a cachectic model. It is a cancer-chemotherapy model resembling the clinical course of treatment for colorectal cancer where treatment would be given after a recent diagnosis. Therefore, we were able to detect changes that occur early in cancer trajectory and follow those changes over clinical course. We acknowledge various limitations of the present study. Effect of tumour and chemotherapy on the other adipose depots needs to be investigated as they all contribute to whole body lipid metabolism. Activity of various proteins and enzymes should be determined in order to provide a broader view of metabolic alterations by anti-neoplastic treatments.

## Conclusions

Diminished expression of proteins involved in mitochondrial function, lipogenesis and β-oxidation contribute to diminished lipid storage capacity of adipose tissue. Adipose tissue was not able to efficiently oxidize fatty acids to provide energy to maintain energy demanding pathways like lipogenesis inside the tissue. This study proposes the need for further evaluations of the effect of early interventions to maintain adipose tissue mitochondrial function in order to maintain adipose tissue mass and function following chemotherapy treatment.

## Additional files

Additional file 1: Lipid metabolism proteins differentially expressed in tumour-bearing animals compared to the reference animals. (XLS 43 kb)

Additional file 2: Differentially expressed proteins in periuterine adipose tissue of animals receiving 2- cycles of chemotherapy compared to tumour-bearing animals. A total of 121 proteins were differentially expressed in periuterine adipose tissue after 2 cycles of chemotherapy compared to the tumour. (DOCX 58 kb)

## Abbreviations

5-FU: 5-fluorouracil
ABCD2: ATP binding cassette subfamily D member 2
ACAA1: acetyl-CoA acyltransferase 1
ACADM: acyl-CoA dehydrogenase, C-4 to C-12 straight chain
ACC: acetyl-CoA carboxylase
ACN: ammonium bicarbonate/acetonitrile
CAT: catalase
COX: cytochrome c oxidase
CPT-11: Irinotecan
CPT2: carnitine palmitoyltransferase 2
DGAT-2: diacylglycerol O-Acyltransferase-2
ECH: enoyl-CoA hydratase
ECHS1: enoyl-CoA hydratase, short chain, 1, mitochondrial
ECI1: enoyl-CoA delta isomerase 1
FAS: Fatty acid synthase
G6PD: Glucose-6-phosphate dehydrogenase
GPD1: Glycerol-3-phosphate dehydrogenase
HSD17B10: Hydroxysteroid (17-beta) dehydrogenase 10
HSL: Hormone sensitive lipase
IDH: Isocitrate dehydrogenase
IPA: Ingenuity pathway analysis
LPL: Lipoprotein lipase
MCP2: Mitochondrial pyruvate carrier 2
MDH2: Malate dehydrogenase 2
MUFA: Monounsaturated fatty acids
NDUFs: NADH dehydrogenase (ubiquinone) subunits
OXPHOS: Oxidative phosphorylation
PDH: Pyruvate dehydrogenase
PGD: Phosphogluconate dehydrogenase
PL: Phospholipid
PPARγ: Peroxisome proliferator-activated receptor gamma
PRDX: Peroxiredoxin
SCD-1: Stearoyl-CoA desaturase-1
SCP-2: Sterol carrier protein 2
SDH: Succinate dehydrogenase
SFA: Saturated fatty acids
SLC25A1: Solute carrier family 25 Member 1
SLC2A4: Solute carrier family 2 member 4
SREBP-1c: Sterol regulatory element binding transcription factor 1
TG: Triglyceride
UQCRC: Coenzyme Q – cytochrome c reductase core protein
VLCAD: Acyl-CoA dehydrogenase, and very long chain
