# Supplementary material for: Chemotherapy diminishes lipid storage capacity of adipose tissue in a preclinical model of colon cancer
Source: Lipids Health Dis. 2017 Dec 19;16:247. doi: 10.1186/s12944-017-0638-8 (PMC5735884; doi:10.1186/s12944-017-0638-8)
Supplement: Supplementary file 2 — Differentially expressed proteins in periuterine adipose tissue of animals receiving 2- cycles of chemotherapy compared to tumour-bearing animals. A total of 121 proteins were differentially expressed in periuterine adipose tissue after 2 cycles of chemotherapy compared to the tumour. (DOCX 58 kb) [file 12944_2017_638_MOESM2_ESM.docx]

| **Accession** | **Gene** | **Protein** | **Fold change** |
| --- | --- | --- | --- |
| P14604 | ECHS1 | Enoyl-CoA hydratase, short chain, 1, mitochondrial | -1.9 |
| P23965 | ECI1 | Enoyl-CoA delta isomerase 1 | -1.6 |
| P04041 | GPX1 | Glutathione peroxidase 1 | -2.0 |
| P23764 | GPX3 | Glutathione peroxidase 3 | -2.2 |
| O70351 | HSD17B10 | Hydroxysteroid (17-beta) dehydrogenase 10 | -1.6 |
| P35704 | PRDX2 | Peroxiredoxin 2 | -1.7 |
| P27791 | PRKACA | Protein kinase cAMP-activated catalytic subunit alpha | -6.3 |
| Q9QY44 | ABCD2 | ATP binding cassette subfamily D member 2 | 6.3 |
| P11915 | SCP2 | Sterol carrier protein 2 | 2.0 |
